# XanFur, a novel Fur protein induced by H_2_O_2_, positively regulated by the global transcriptional regulator Clp and required for the full virulence of *Xanthomonas oryzae* pv. *oryzae* in rice

**DOI:** 10.1128/spectrum.01187-23

**Published:** 2023-10-13

**Authors:** Yu-Qiang Zhang, Xiao-Yan Song, Fengquan Liu

**Affiliations:** 1 Institute of Plant Protection, Jiangsu Academy of Agricultural Sciences, Nanjing, Jiangsu, China; 2 State Key Laboratory of Microbial Technology, Shandong University, Qingdao, Shandong, China; Pennsylvania State University, State College, Pennsylvania, USA

**Keywords:** bacterial leaf blight, *Xanthomonas oryzae *pv. *oryzae*, *xanfur*, virulence, Clp

## Abstract

Bacterial leaf blight caused by *Xanthomonas oryzae* pv. *oryzae* (*Xoo*) is one of the most destructive diseases in rice. However, the pathogenic mechanisms of *Xoo* have not been fully understood. In this study, we identified a novel ferric uptake regulator (Fur) protein, XanFur, in *Xoo,* which is conserved among the major pathogens of *Xanthomonas* species, but has no obvious sequence identity with reported Fur proteins. Gene deletion indicated that *xanfur* is required for the full virulence of *Xoo* in rice. The expression of *xanfur* was significantly induced by 0.1 mM H_2_O_2_ and its deletion caused a significant decrease in the tolerance of *Xoo* to H_2_O_2_, suggesting that *xanfur* is likely involved in the resistance of *Xoo* to the oxidative stress caused by H_2_O_2_ in rice. The loss of *xanfur* also caused significant reduction in extracellular polysaccharide production, biofilm formation, and cell motility, suggesting that *xanfur* may be also involved in regulating these virulence determinants of *Xoo*. Furthermore, both *in vivo* and *in vitro* experimental results demonstrated that the global transcriptional regulator Clp positively regulated the expression of *xanfur* by directly binding to its promoter region, especially in the presence of 0.1 mM H_2_O_2_, presenting the cellular regulatory pathway for the expression of this gene. These results not only contribute to a better understanding of the interaction mechanisms between rice and *Xoo*, but also provide reference to developing high-effective bactericides by targeting to the Fur protein in *Xoo* to control bacterial leaf blight in rice.

**IMPORTANCE:**

Although *Xanthomonas oryzae* pv. *oryzae* (*Xoo*) has been found to be a bacterial pathogen causing bacterial leaf blight in rice for many years, the molecular mechanisms of the rice-*Xoo* interaction has not been fully understood. In this study, we found that XanFur of *Xoo* is a novel ferric uptake regulator (Fur) protein conserved among major pathogenic *Xanthomonas* species. XanFur is required for the virulence of *Xoo* in rice, and likely involved in regulating the virulence determinants of *Xoo*. The expression of *xanfur* is induced by H_2_O_2_, and positively regulated by the global transcriptional regulator Clp. Our results reveal the function and regulation of the novel virulence-related Fur protein XanFur in *Xoo,* providing new insights into the interaction mechanisms of rice-*Xoo*.

## INTRODUCTION

As the most important staple food, rice provides approximately 23% of calories for more than half of the world population, but its production is threatened by a variety of diseases, such as rice blast, rice sheath blight, rice stripe, and bacterial leaf blight (1 – 4). Bacterial leaf blight caused by the bacterial pathogen *Xanthomonas oryzae* pv. *oryzae* (*Xoo*) is one of the most destructive diseases in rice, resulting in up to 50% yield loss in the major rice-growing countries (5). *Xoo* usually infects rice through leaf hydathodes or wound sites and utilizes the nutritional sources in rice for its generation, resulting in tissue necrosis and wilting (6). The interaction between *Xoo* and rice has become a vital working model to elucidate how pathogens weaken or block the immune responses of host plants (7).

Recently, plenty of works has been done to study the molecular mechanisms of the rice-pathogens interaction. For example, the fungal pathogens *Magnaporthe oryzae* and *Rhizoctonia solani* depend on appressorium to enter the rice tissues, and produce effectors and RS toxin which is a mixture of N-acetyl glucosamine, N-acetyl galactosamine, glucose, and mannose to cause rice blast and rice sheath blight, respectively (8, 9). On the other hand, rice develops varieties of immune responses to prevent the infection of pathogens. For example, rice generates reactive oxygen species (ROS) to cause oxidative stress to the infected pathogens. It has been reported that the H_2_O_2_ burst regulated by RbohB (respiratory burst oxidase protein B-like) is required for the resistance of rice to the fungal pathogen *M. oryzae* (10). It was also reported that H_2_O_2_ is toxic to bacteria and causes damages to proteins and cell membranes (11). Therefore, the pathogens need to overcome the oxidative stress that resulted from H_2_O_2_ to cause rice diseases. Works on the pathogenic mechanisms of *Xoo* have revealed that extracellular polysaccharides (EPS) protect *Xoo* against biotic or abiotic stresses, biofilm enhances the attachment of *Xoo* to different surfaces, cell motility allows *Xoo* to avoid toxic compounds, and type III secretion system contributes to the secretion of effectors into rice tissues, all of which are required determinants for the full virulence of *Xoo* in rice (12 – 15). However, the molecular mechanisms of the rice-*Xoo* interaction have not been fully understood. For example, the mechanisms of which *Xoo* responses to the oxidative stress caused by H_2_O_2_ in rice still remain unknown.

In plants, while the metal element availability is likely a limiting factor to the growth of pathogens, excess intracellular metal element is also toxic to themselves. Therefore, the metal homeostasis is crucial for the survival of pathogens and plays important roles in the plant-pathogen interaction (16, 17). The Fur (ferric uptake regulator) family proteins are responsible for the homeostasis of the metal elements in pathogens, which have been divided into several groups, including the Fur, Zur (Zinc uptake regulator), manganese uptake regulator, nickel uptake regulator, and peroxide stress response regulator (18). In addition to their functions in regulating metal homeostasis, these Fur family proteins also regulate the expression of genes involved in the virulence and resistance to oxidative stress in pathogens. As the founding member of the Fur family proteins, the Fur proteins have been studied widely to understand its relationship with the virulence of pathogens. For example, the loss of the Fur protein from *Xanthomonas campestris* pv. *campestris* caused significant decrease in its virulence in Chinese cabbage and its tolerance to H_2_O_2_ (19). The loss of the Fur protein also caused significant decrease in the virulence of *Pectobacterium carotovorum* subsp. *brasiliense* in potato, of *Acidovorax citrulli* in watermelon, and of *Xanthomonas vesicatoria* in tomato, and in the EPS production, biofilm formation, and cell motility of these pathogens (20 – 22). Although many Fur proteins have been found in pathogens with the development of biochemical, molecular genetics, and wide-genome assays, our understanding on their functions in regulating the virulence of the pathogens is still limited.

As for the Fur proteins in *Xoo*, Subramoni and Sonti reported that a Fur protein (accession no. AF146830) was required for the full virulence of *Xoo* in rice (23). In addition, the gene *PXO_RS06520* (previously named gene *PXO_04819*) of *Xoo* was predicted to encode a Fur family transcriptional regulator, and interacting transcriptomes revealed that its expression in *Xoo* was significantly induced after the inoculation of *Xoo* in rice (24). However, the function of gene *PXO_RS06520* in regulating the virulence of *Xoo* in rice still lacks investigation.

In this study, we found that gene *PXO_RS06520* (named *xanfur* in this study) encodes a Fur protein (XanFur) that was highly conserved among the major pathogens of *Xanthomonas* species and its expression was significantly induced by H_2_O_2_. The loss of *xanfur* resulted in significant decrease in the full virulence of *Xoo* in rice, and in its EPS production, biofilm formation, cell motility, and tolerance to H_2_O_2_. Moreover, we found that the global transcriptional regulator Clp (cAMP receptor protein-like protein) positively controlled the expression of *xanfur* by directly binding to its promoter region. The results indicated that XanFur is a novel H_2_O_2_-induced Fur protein involved in the virulence of *Xoo*.

## RESULTS AND DISCUSSION

### XanFur encoded by *PXO_RS06520* of *Xoo* represents a new Fur family transcriptional regulator

**Fig 1:**
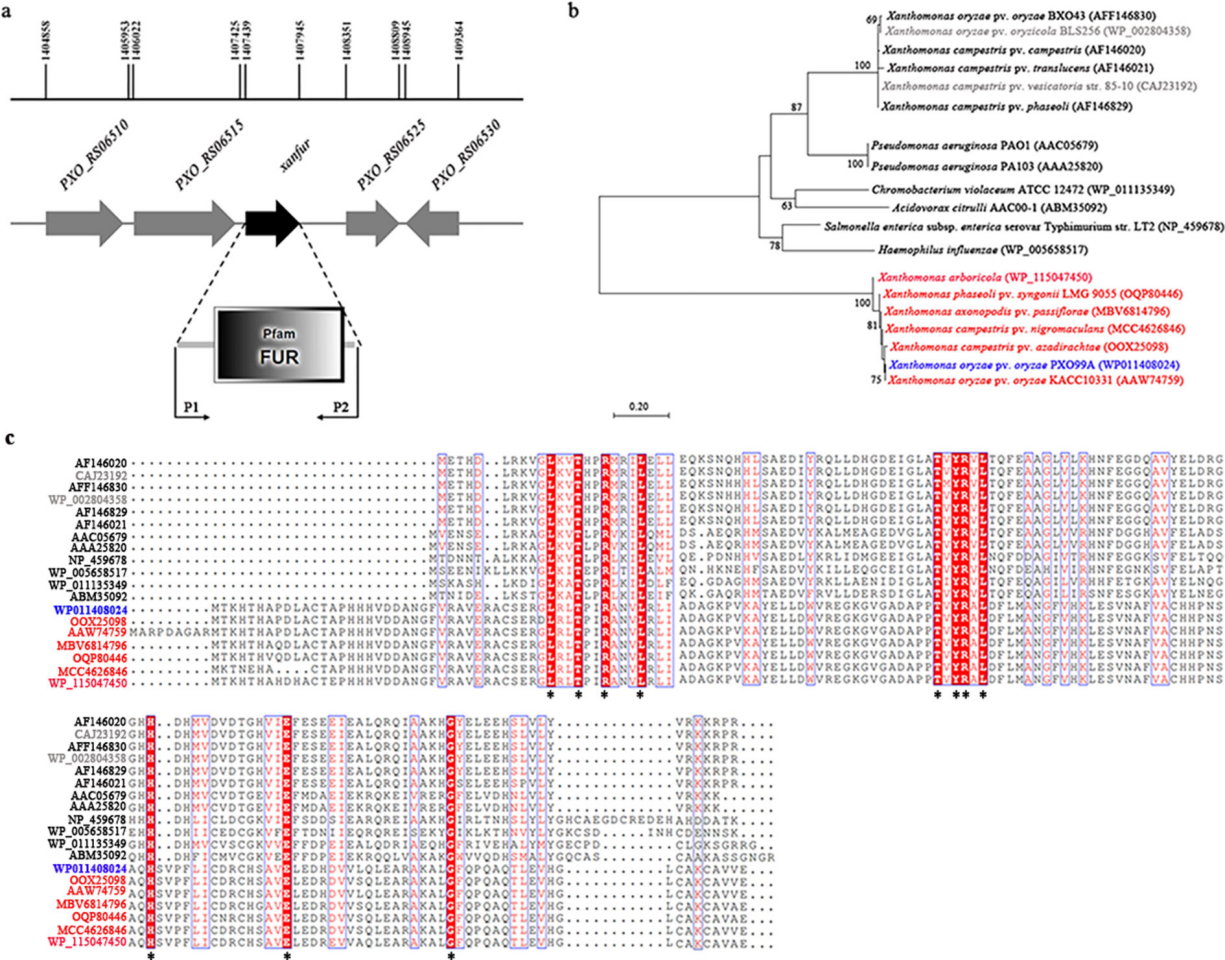
Bioinformatic analysis of XanFur. (**a**) Schematic diagram of *xanfur* in the genome of *Xoo*. The arrows indicate the location, length, and orientation of the open reading frames (ORFs). The middle element shows the conserved domain of XanFur. The lower element shows primers P1 and P2 used in amplication of *xanfur* by using PCR. (**b**) Amino acid sequence alignment of XanFur with 18 Fur proteins from major pathogens. The blue-colored accession number represents the XanFur protein from *Xoo*. The red-colored accession numbers represent the putative Fur proteins from *Xanthomonas* species. The gray-colored accession numbers represent the Fur proteins reported to be not required for virulence of pathogens. The black-colored accession numbers represent the Fur proteins reported to be required for the virulence of pathogens. The pathogens containing the 18 Fur proteins are shown in (**c**). (**c**) Phylogenetic analysis of XanFur with 18 Fur proteins from major pathogens. The phylogenetic tree was built by using the neighbor-joining method with a Jones-TaylorThornton matrix-based model. The scale for the branch length was shown below the tree. Accession numbers are colored as in (**b**).

The encoding region of gene *PXO_RS06520* in the genome of *Xoo* is from 1407439 to 1407945, the genomic sequence length of *PXO_RS06520* is 507 bp, and the amino acids size is 169 aa. Meanwhile, *PXO_RS06520* encodes a protein containing only a Fur domain based on SMART prediction (http://smart.embl-heidelberg.de/), suggesting that gene *PXO_RS06520* encodes a Fur protein (Fig. 1a).

Amino acid sequence alignment showed that the protein encoded by gene *PXO_RS06520* exhibited high sequence identity to the putative Fur proteins from some *Xanthomonas* pathogens, including *Xoo* KACC10331 (AAW74759, 100%), *X. campestris* pv. *azadirachtae* (OOX25098, 98.21%), *X. phaseoli* pv. *syngonii* LMG 9055 (OQP80446, 96.43%), *X. campestris* pv. *nigromaculans* (MCC4626846, 98.73%), *X. axonopodis* pv. *passiflorae* (MBV6814796, 97.02%), and *X. arboricola* (WP_115047450 , 95.83%). The result indicated that gene *PXO_RS06520* likely encodes a Fur protein highly conserved among the major pathogens of *Xanthomonas* species, which was named XanFur in this study.

In contrast, XanFur exhibited quite low sequence identity to the Fur proteins which have been reported in *Xanthomonas* pathogens, including *X. oryzae* pv. *oryzicola* BLS256 (WP_002804358, 14.71%), *X. campestris* pv. *translucens* (AF146021, 13.24%), *X. campestris* pv. *vesicatoria* str. 85–10 (CAJ23192, 14.71%), *X. campestris* pv. *phaseoli* (AF146829, 14.71%) (25), *X. campestris* pv. *campestris* (AF146020, 14.71%) (19), and *Xoo* BXO43 (AF146830, 13.97%) (23), as well as those required for the virulence of other pathogens, including *Chromobacterium violaceum* ATCC 12472 (WP_011135349, 15.38%) (26), *Salmonella enterica* subsp. *enterica* serovar Typhimurium str. LT2 (NP_459678, 15.33%) (27), *A. citrulli* AAC00-1 (ABM35092, 17.93%) (22), *Pseudomonas aeruginosa* PAO1 (AAC05679, 14.93%) (28), *P. aeruginosa* PA103 (AAA25820, 17.16%) (29), and *Haemophilus influenzae* 86-028NP (AAX87247, 14.38%) (30) (Fig. 1b). Consistently, phylogenetic analysis showed that XanFur was clustered with some putative Fur proteins from *Xanthomonas* pathogens, forming a branch separate from the reported Fur proteins (Fig. 1c).

These data indicated that XanFur and its homologs represent a novel Fur protein highly conserved among the major pathogens of *Xanthomonas* species, whose function is yet unknown. The gene *PXO_RS06520* encoding XanFur was named *xanfur* hereafter for convenience.

### The expression of *xanfur* is significantly induced by H_2_O_2_

**Fig 2:**
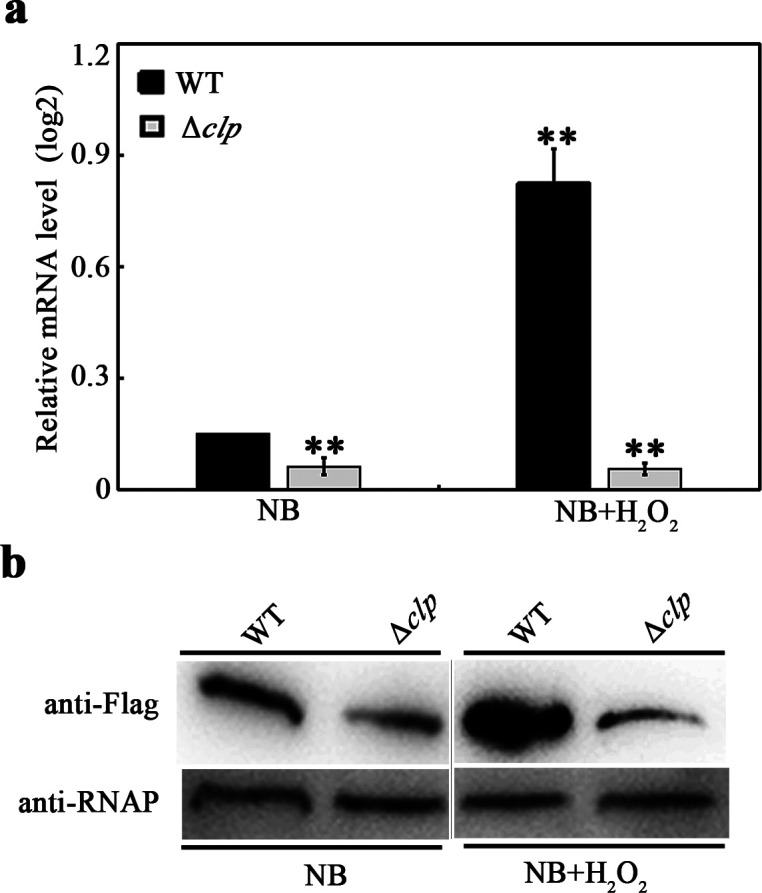
The effect of H_2_O_2_ on the expression of XanFur in *Xoo* strains WT and Δ*clp*. (**a**) The relative mRNA level of *xanfur* in *Xoo* strains WT and Δ*clp* detected by using qRT-PCR. (**b**) The representative detection of XanFur expressed in *Xoo* strains WT and Δ*clp* by using western blot. The upper bands corresponding to the predicted size of *xanfur*-Flag fusion were detected by anti-Flag monoclonal antibody, and the lower bands corresponding to the predicted size of RNA polymerase α-subunit were detected by the specific antibody anti-RNAP. WT, the wild-type *Xoo* strain PXO99^A^. Δ*clp*, the *clp* deletion mutant strain of WT. Strains were cultured in nutrient broth (NB) liquid medium with or without 0.1 mM H_2_O_2_ to OD_600_ = 1.0. The experiment was independently repeated three times. Values were the means ± SDs from three independent experiments. The asterisks above the error bars indicated significant differences compared with the expression in strain WT which was incubated without 0.1 mM H_2_O_2_ (*t*-test, **P < 0.01).

It has been reported that rice can form H_2_O_2_ burst to resist fungal pathogen (10). To investigate whether the expression of *xanfur* in *Xoo* is induced by exterior H_2_O_2_, quantitative real-time PCR (qRT-PCR) and western blot were performed. The result of qRT-PCR showed that, compared to that in *Xoo* untreated with H_2_O_2_, the relative mRNA level of *xanfur* was significantly increased approximately sixfold in *Xoo* treated with 0.1 mM H_2_O_2_ (Fig. 2a). Consistently, western blot analysis showed that the protein band of XanFur was significantly increased 1.9-fold in *Xoo* treated with 0.1 mM H_2_O_2_, compared to that in *Xoo* untreated with 0.1 mM H_2_O_2_. Meanwhile, the protein band of the internal control RNA polymerase (RNAP) exhibited no remarkable increase in *Xoo* treated with 0.1 mM H_2_O_2_, compared to that in *Xoo* untreated with 0.1 mM H_2_O_2_ (Fig. 2b). These results indicated that the expression of *xanfur* in *Xoo* was significantly induced by H_2_O_2_.

In response to pathogen infection, rice can produce a high level of ROS, which is toxic to pathogens (31), and pathogens in turn can use ROS as signals to up-regulate the expression of virulence-associated genes (32). So far, it is yet unknown which genes in *Xoo* are induced by ROS. Since rice can form H_2_O_2_ burst to resist pathogen (10), pathogens may also respond to the increased H_2_O_2_ and up-regulate the expression of virulence-associated genes to cope with the oxidative stress caused by H_2_O_2_ in their interaction with rice. Here, our result showed that the expression of *xanfur* in *Xoo* can be significantly induced by H_2_O_2_, implying that *xanfur* may be a virulence-related gene of *Xoo*.

### XanFur is required for the full virulence of *Xoo* in rice

**Fig 3:**
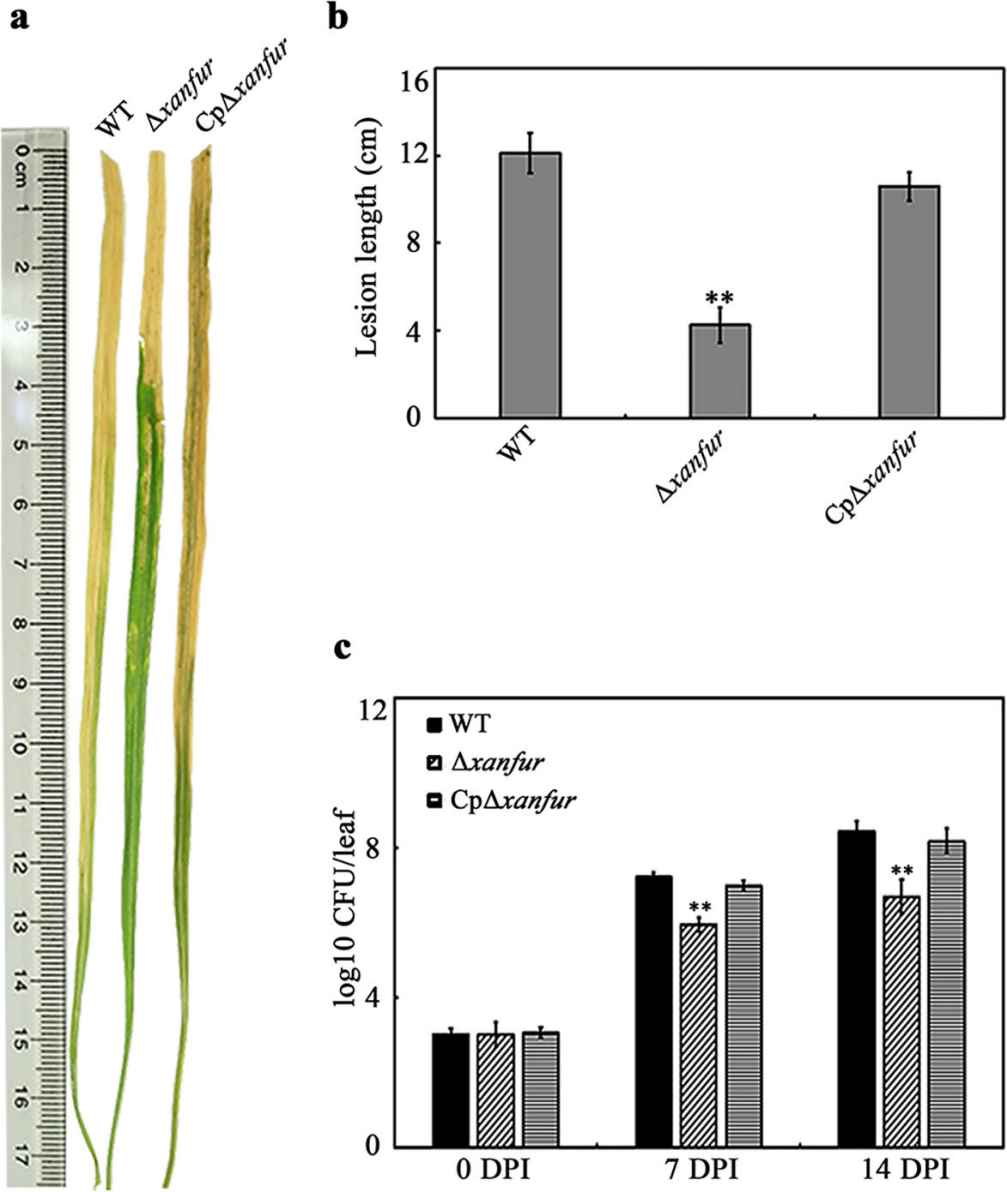
The effect of *xanfur* deletion on the full virulence of *Xoo* in rice. (**a**) The representative lesion lengths in rice leaves inoculated with the *Xoo* strains WT, Δ*xanfur,* and CpΔ*xanfur* after 14 days. (**b**) The calculated lesion lengths in rice leaves inoculated with the *Xoo* strains WT, Δ*xanfur,* and CpΔ*xanfur* after 14 days. (**c**) The investigated bacterial population in rice leaves inoculated with the *Xoo* strains WT, Δ*xanfur,* and CpΔ*xanfur* after 0, 7, and 14 days. WT, the wild-type *Xoo* strain PXO99^A^. Δ*xanfur*, the *xanfur* deletion mutant strain of WT. CpΔ*xanfur*, the Δ*xanfur* complementary strain of WT. DPI, day post-inoculation. The experiment was independently repeated three times. Values are the means ± SDs from three independent experiments. The asterisks above the error bars indicate significant differences compared with the wild-type strain (*t*-test, **P < 0.01).

To investigate whether XanFur is involved in the virulence of *Xoo* in rice, a *xanfur* deletion mutant strain (Δ*xanfur*) of *Xoo* and its complementary strain (CpΔ*xanfur*) were successfully constructed (Fig. S1a). The deletion of *xanfur* caused no significant difference in the growth of wild type (WT) and Δ*xanfur* in both NB liquid medium and MMX (a minimal medium without ammonium sulphate as the nitrogen source) liquid medium (Fig. S1b and c), indicating that *xanfur* is not necessary for the growth of *Xoo*. The effect of *xanfur* on the full virulence of *Xoo* was then analyzed by comparing the virulence of strains WT, Δ*xanfur,* and CpΔ*xanfur* in rice. The lesion length caused by the inoculation of WT was approximately 12 cm, but that caused by the inoculation of Δ*xanfur* was only approximately 4 cm. The lesion length caused by the inoculation of CpΔ*xanfur* displayed a similar level with that caused by WT (Fig. 3a and b). In addition, the cell population of Δ*xanfur* in rice leaf was significantly lower than that of WT or CpΔ*xanfur* in rice leaf 7 and 14 days post-inoculation (Fig. 3c). These data demonstrated that XanFur is required for the full virulence and the colonization of *Xoo* in rice, suggesting that *xanfur* is a virulence-related gene, and that XanFur is likely involved in the regulation of the virulence of *Xoo* to rice.

Some Fur proteins have been reported to be involved in regulating the virulence of plant bacterial pathogens, such as those in *Pectobacterium atrosepticum* (32), *A. citrulli* AAC00-1 (22), and *S. enterica serovar* Typhimurium (27, 33). In recent years, some Fur proteins have also been reported to be required for the virulence of *Xanthomonas* species, including those in *X. vesicatoria*, *X. campestris* pv. *phaseoli,* and *X. campestris* pv. *campestris* (19, 20, 25). Although XanFur exhibits no significant sequence identity to the reported Fur proteins (Fig. 1b), our results indicate that XanFur is also required for the virulence of *Xoo* in rice.

It has been shown that the reduced virulence of *Xoo* in rice was related to its growth deficiency caused by the deletion of some genes, such as *xrvC*, *fur,* and *rnr* (23, 34, 35). However, the loss of *xanfur* caused no significant deficiency in the growth of *Xoo* in both NB liquid medium and MMX liquid medium (Fig. S1b and c), but resulted in attenuated virulence in rice. This implies that *xanfur* may have different pathways in regulating the virulence of *Xoo* in rice from the reported genes *xrvC*, *fur,* and *rnr*, which, however, awaits further investigation.

### XanFur is essential for the tolerance of *Xoo* to H_2_O_2_

**Fig 4:**
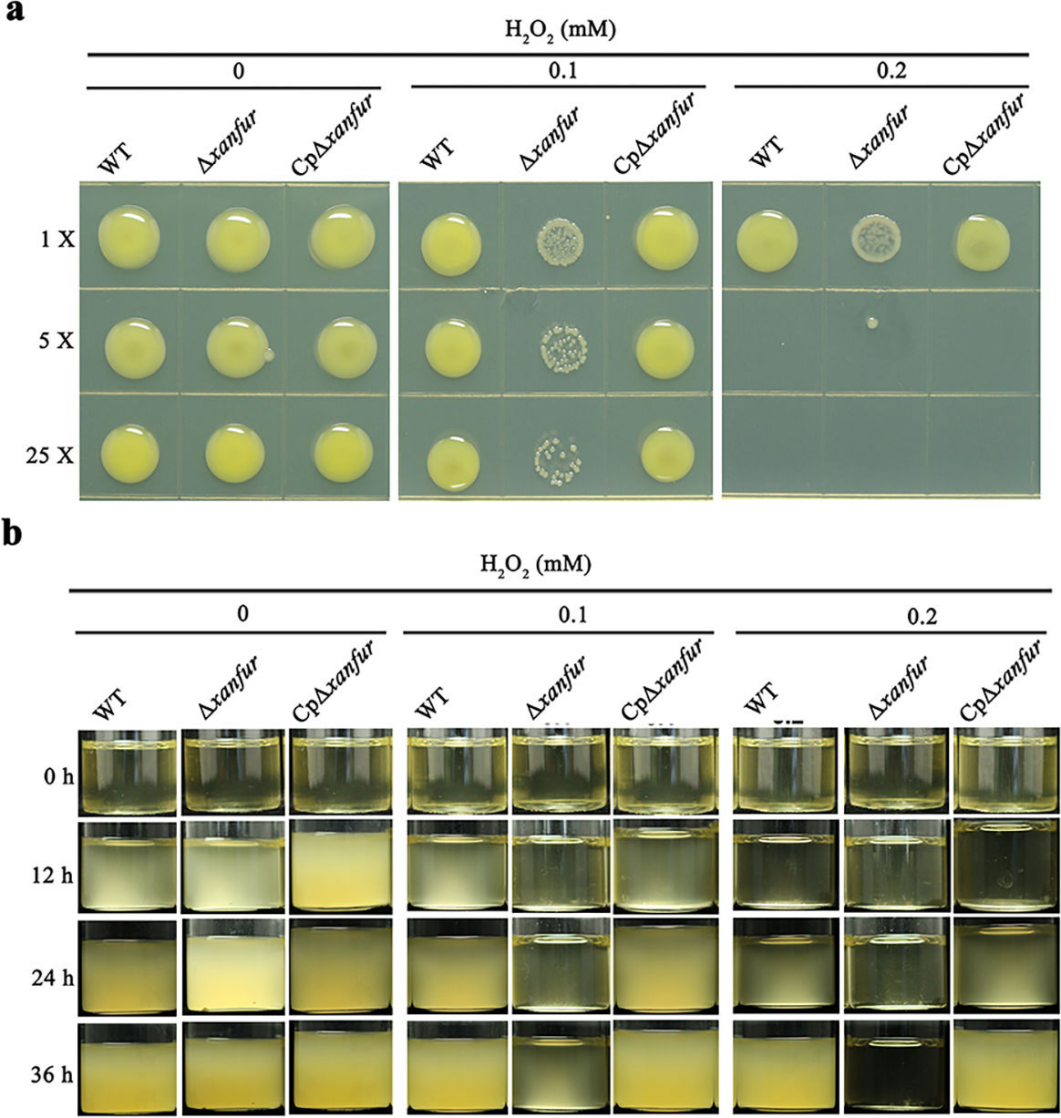
The effect of *xanfur* deletion on the tolerance of *Xoo* to H_2_O_2_. (**a**) The representative growth of *Xoo* strains WT, Δ*xanfur,* and CpΔ*xanfur* on NB solid medium plates with 0 mM, 0.1 mM, or 0.2 mM H_2_O_2_ for 2 days. 1×, 5×, and 25×, the bacterial suspension was diluted 1-, 5-, and 25-fold. (**b**) The representative growth of *Xoo* strains WT, Δ*xanfur,* and CpΔ*xanfur* in 8 mL NB liquid medium with 0 mM, 0.1 mM, or 0.2 mM H_2_O_2_ for 36 h. WT, the wild-type strain of *Xoo* PXO99^A^. Δ*xanfur*, the *xanfur* deletion mutant strain of WT. CpΔ*xanfur*, the Δ*xanfur* complementary strain of WT. The experiment was independently repeated three times.

Since the expression of *xanfur* in *Xoo* was induced by H_2_O_2_ (Fig. 2), XanFur may be involved in the tolerance of *Xoo* to H_2_O_2_. To support this hypothesis, we compared the H_2_O_2_ tolerance of strains WT, Δ*xanfur,* and CpΔ*xanfur*. When cultivated on NB solid medium or in NB liquid medium without H_2_O_2_, strains WT, Δ*xanfur,* and CpΔ*xanfur* showed similar growth. However, when 0.1 mM or 0.2 mM H_2_O_2_ was presented in NB media, the growth of Δ*xanfur* was significantly inhibited, but that of WT or CpΔ*xanfur* did not (Fig. 4a and b), indicating that the H_2_O_2_ tolerance of Δ*xanfur* was much weaker than that of WT. Thus, the deletion of *xanfur* resulted in a significant decrease in the H_2_O_2_ tolerance of *Xoo*, suggesting that XanFur plays a role in the tolerance of *Xoo* to H_2_O_2_.

The ability to adapt to adverse environmental stresses is essential for the infection of pathogens. Some Fur proteins have been shown to be able to affect the tolerance of *Xanthomonas* species to H_2_O_2_. For example, the Fur protein (accession no. WP_005988392) affected the H_2_O_2_ tolerance of *X. campestris* pv. *campestris* in Chinese cabbage (19), and the Fur protein (accession no. AF146830) affected the H_2_O_2_ tolerance of *Xoo* in rice (23). The deletion of *fur* from *A. citrulli* AAC00-1 (22) and *P. aeruginosa* PA103 (29) caused an increased sensitivity of these pathogens to H_2_O_2_. Consistent with these Fur proteins, XanFur is also required for the tolerance of *Xoo* to H_2_O_2_, which suggests that XanFur is likely involved in the resistance of *Xoo* to the oxidative stress caused by H_2_O_2_ in rice.

### XanFur is essential for some important virulence determinants of *Xoo*

It has been shown that EPS, biofilm, and cell motility are required determinants for the full virulence of *Xoo* to rice (12). To investigate whether XanFur is involved in the regulation of these virulence determinants of *Xoo*, we compared the EPS production, biofilm formation, and cell motility of strains WT, Δ*xanfur,* and CpΔ*xanfur*.

**Fig 5:**
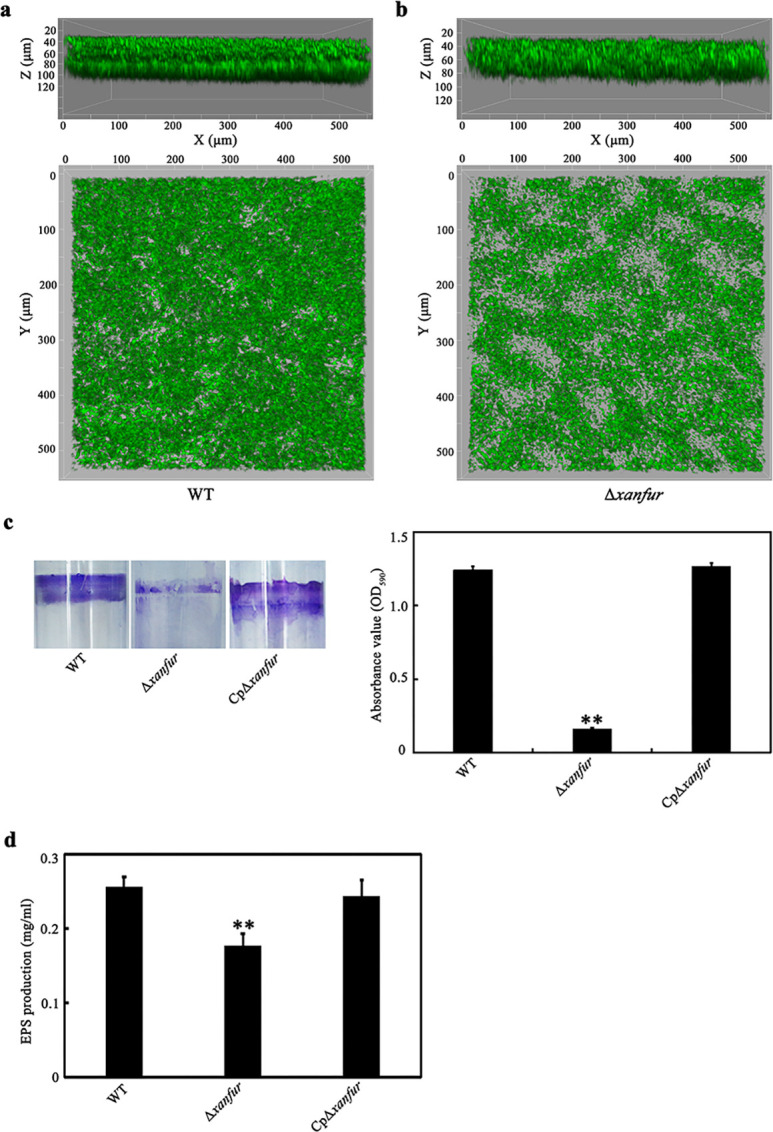
The effect of *xanfur* deletion on the biofilm formation and EPS production of *Xoo*. (**a and b**) The representative biofilm formed by *Xoo* strains WT and Δ*xanfur*. The representative thickness of the biofilm (up, from the angles of X- and Z-axis) and the representative organized structure of the biofilm (down, from the angles of X and Y axis) were analyzed by using confocal laser scanning microscopy. Images were obtained by using a 20× objective. (**c**) The representative biofilm formed by *Xoo* strains WT, Δ*xanfur,* and CpΔ*xanfur* on polystyrene tubes stained with crystal violet (left) and the calculated biofilm production (right). The values of OD_590_ stand for biofilm production. (**d**) The EPS production of *Xoo* strains WT, Δ*xanfur,* and CpΔ*xanfur* which were cultured in NB liquid medium for 5 days. WT, the wild-type strain of *Xoo* PXO99^A^. Δ*xanfur*, the *xanfur* deletion mutant strain of WT. CpΔ*xanfur*, the Δ*xanfur* complementary strain of WT. The experiment was independently repeated three times. Values are the means ± SDs from three independent experiments. The asterisks above the error bars indicate significant differences compared with the wild-type strain (*t*-test, **P < 0.01).

Biofilm formation is associated with the attachment of pathogens to different environmental surfaces (36). Thus, we evaluated the effect of *xanfur* deletion on the biofilm formation of *Xoo*. The confocal laser scanning microscope observation showed that the thickness of the biofilm formed by strain WT was approximately 80 µM with a well-organized structure, but that formed by strain Δ*xanfur* was only approximately 60 µM with a less organized structure (Fig. 5a and b). Consistently, the biofilm formed by Δ*xanfur* (approximately 0.2 of optical density at 590 nm [OD_590_]) on the surface of a polystyrene tube was significantly smaller than that formed by WT (approximately 1.25 of OD_590_) or CpΔ*xanfur* (approximately 1.32 of OD_590_) (Fig. 5c). Thus, the loss of *xanfur* caused significant decrease in biofilm formation of *Xoo*.

EPS, a virulence determinant of *Xoo*, is involved in the biofilm formation of *Xoo* (12). Therefore, we further investigated the effect of *xanfur* deletion on the EPS production of *Xoo*. Strains WT, Δ*xanfur,* and CpΔ*xanfur* were cultured in NB liquid medium to compare their EPS production. The results showed that the EPS produced by Δ*xanfur* (approximately 0.18 mg/mL) was significantly lower than that produced by WT or CpΔ*xanfur* (both approximately 0.25 mg/mL) (Fig. 5d), indicating that the deletion of *xanfur* significantly weakened the EPS production of *Xoo*.

**Fig 6:**
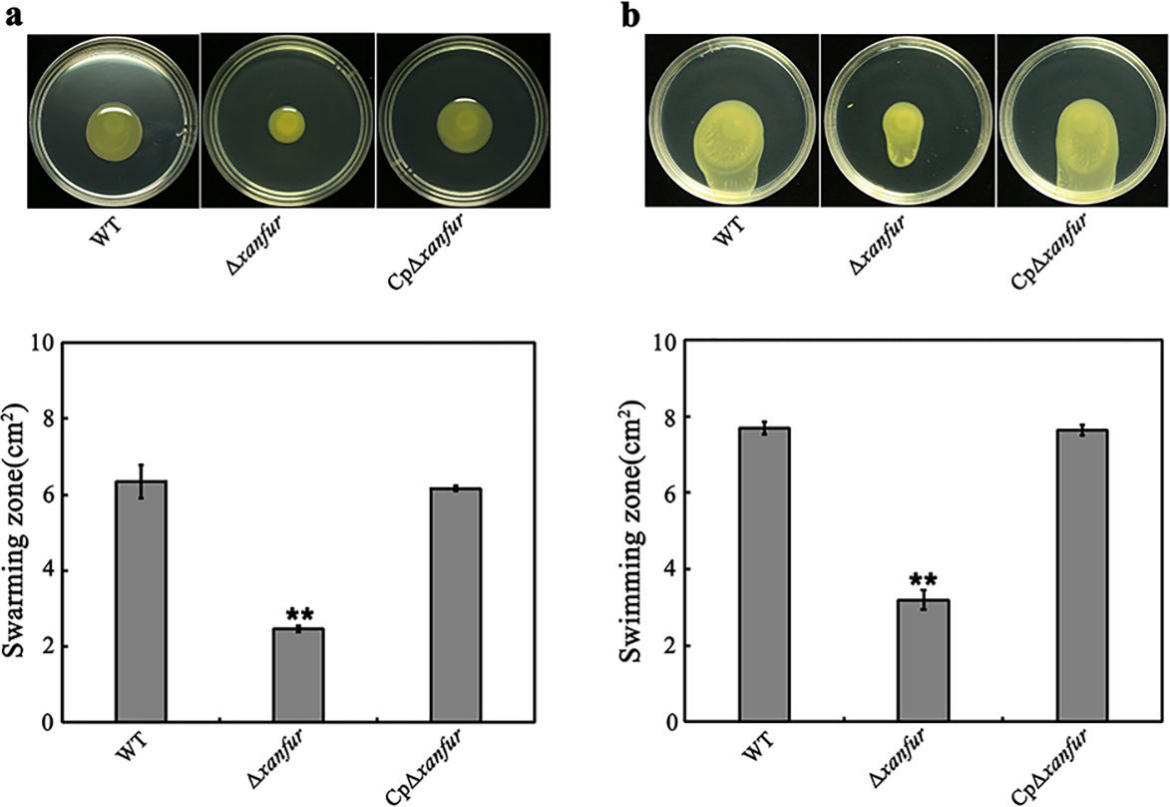
The effect of *xanfur* deletion on the cell motility of *Xoo*. (**a**) The representative swarming zone (up) and the calculated area of the swarming zone (down) produced by *Xoo* strains WT, Δ*xanfur,* and CpΔ*xanfur* which were cultured on NB solid medium plates containing 0.6% agar for 3 days. (**b**) The representative swimming zone (up) and the calculated area of the swimming zone (down) produced by *Xoo* strains WT, Δ*xanfur,* and CpΔ*xanfur* which were cultured on NB solid medium plate containing 0.3% agar for 2 days. WT, the wild-type strain of *Xoo* PXO99^A^. Δ*xanfur*, the *xanfur* deletion mutant strain of WT. CpΔ*xanfur*, the Δ*xanfur* complementary strain of WT. The experiment was independently repeated three times. Values are the means ± SDs from three independent experiments. The asterisks above the error bars indicate significant differences compared with the wild-type strain (*t*-test, **P < 0.01).

Cell motility is another important virulence determinant for *Xanthomonas* species to avoid themselves from the unfavorable environments. To analyze the effect of *xanfur* deletion on the cell motility of *Xoo*, we compared the swimming and swarming motility of strains WT, Δ*xanfur,* and CpΔ*xanfur*. As shown in Fig. 6a, the swarming zone produced by Δ*xanfur* (approximately 2.46 cm^2^) on the NB solid medium plate with 0.6% agar was significantly smaller than those produced by WT (approximately 6.34 cm^2^) and CpΔ*xanfur* (approximately 6.18 cm^2^). Similarly, the swimming zone produced by Δ*xanfur* (approximately 3.20 cm^2^) was also significantly smaller than those produced by WT (approximately 7.69 cm^2^) and CpΔ*xanfur* (approximately 7.59 cm^2^) (Fig. 6b). These data indicated that the loss of *xanfur* significantly decreased the swimming and swarming motility of *Xoo*.

Altogether, these results indicated that XanFur is essential for the virulence-related determinants of *Xoo*, including EPS production, biofilm formation, and cell motility, suggesting that this regulator is involved in the regulation of these virulence-related determinants, directly or indirectly.

It has been shown that the deletion of *fur* from *P. aeruginosa* PAO1 had no effect on its biofilm formation (28). However, the deletion of *fur* from *X. vesicatoria* (20)*, A. citrulli* AAC00-1 (22), or *C. violaceum* ATCC 12472 (26) not only caused significant decrease in biofilm formation, but also significant decrease in EPS production and cell motility of these strains, suggesting that the *fur*-encoding proteins are involved in the regulation of these virulence-related determinants in these strains. Similarly, the deletion of *xanfur* from *Xoo* also caused significant decrease in EPS production, biofilm formation, and cell motility. Thus, XanFur also plays an important role in regulating the virulence determinants of *Xoo*. However, how XanFur and other Fur proteins regulate the virulence determinants of these pathogens awaits further investigation.

### Clp positively regulates the expression of *xanfur* by directly binding to its promoter region

Clp is a conserved global regulator among the pathogenic *Xanthomonas* species. It has been shown that the expression of some virulence-related genes can be regulated by Clp (37, 38). However, no *fur* gene has been reported to be regulated by Clp. To investigate whether the expression of *xanfur* could be regulated by Clp in *Xoo*, qRT-PCR and western blot were performed. The result of qRT-PCR showed that when strains were cultured in NB liquid medium without H_2_O_2_, the relative mRNA level of *xanfur* in Δ*clp* was decreased approximately threefold compared to that in WT. However, when strains were cultured in NB liquid medium containing 0.1 mM H_2_O_2_, the relative mRNA level of *xanfur* in Δ*clp* was decreased approximately 18-fold compared with that in WT (Fig. 2a). Consistently, the result of western blot also showed that the protein band of XanFur was decreased 2.3-fold in Δ*clp* cultured in NB liquid medium without H_2_O_2_, and 4.4-fold in Δ*clp* cultured in the presence of 0.1 mM H_2_O_2_, compared to that in WT with the same culture condition. Meanwhile, the protein band of the internal control RNAP exhibited no remarkable reduction in Δ*clp* cultured with or without 0.1 mM H_2_O_2_, compared to that in WT with the same culture condition (Fig. 2b). These results indicated that the expression of *xanfur* was positively regulated by Clp, especially in the presence of 0.1 mM H_2_O_2_.

It has been shown that the deletion of *clp* from *Xoo* resulted in decrease in cell motility, EPS production, and virulence in rice (38). To further determine whether *xanfur* is a downstream component of *clp*, we constructed the *xanfur* deletion mutant strain of Δ*clp* (Δ*xanfur&clp*) and its complementary strain [CpΔ*xanfur*(Δ*clp*)] (Fig. S1a), and compared the growth, cell motility, EPS production, and virulence of strains WT, Δ*clp*, Δ*xanfur&clp,* and CpΔ*xanfur*(Δ*clp*). The growth of strains Δ*clp*, Δ*xanfur&clp,* and CpΔ*xanfur*(Δ*clp*) in NB liquid medium and MMX liquid medium was only a little weaker compared with that of WT under the same conditions (Fig. S1b and c). While strains Δ*clp*, Δ*xanfur&clp,* and CpΔ*xanfur*(Δ*clp*) all showed a significantly decreased virulence in rice compared with that caused by strain WT, the virulence caused by Δ*xanfur&clp* was not significantly weaker than that caused by Δ*clp*, indicating that the deletion of *xanfur* from Δ*clp* did not cause a further significant decrease in the virulence (Fig. S2). In addition, the deletion of *xanfur* from Δ*clp* also caused no significant decrease in biofilm formation, EPS production, or cell motility (Fig. S3). All the data demonstrated that *xanfur* is a downstream component of Clp in *Xoo*.

**Fig 7:**
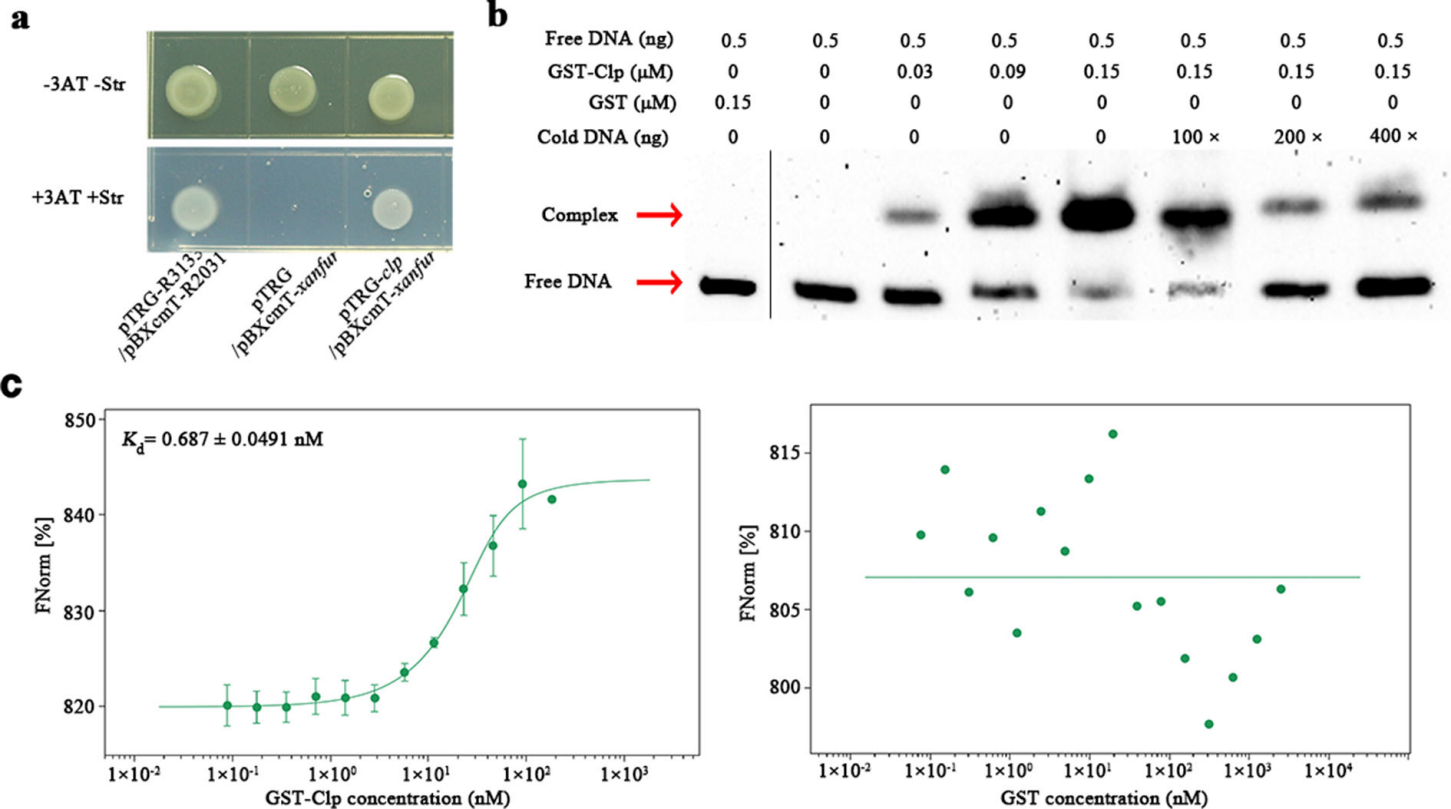
Detection of the interaction between Clp and the promoter of *xanfur in vivo* and *in vitro*. (**a**) The representative *in vivo* interaction between Clp and the promoter of *xanfur* analyzed by using B1H in *E. coli*. pTRG-R2031/pBXcmT-R3133, the cells containing plasmids pBXcmT-R2031 and pTRG-R3133 used as the positive control. pTRG/pBXcmT-*xanfur*, the cells containing the plasmid pBXcmT-*xanfur* and the empty plasmid pTRG used as the negative control. pTRG-*clp*/pBXcmT-*xanfur*, the cells containing plasmids pTRG-*clp* and pBXcmT-*xanfur*. −3-AT − Str, the M9-based solid medium plates without 3-amino-1,2,4-triazole and streptomycin. +3-AT + Str, the M9-based solid medium plates with 5 mM/L 3-amino-1,2,4-triazole and 8 µg/mL streptomycin. (**b**) The representative *in vitro* interaction between Clp and the promoter of *xanfur* analyzed by using electrophoretic mobility shift assay. The arrows indicate the complex bands of free DNA with Clp (up) and the bands of free DNA (down). Free DNA, the biotin-labeled promoter of *xanfur*. Complex, Clp interacting with the biotin-labeled promoter of *xanfur*. Cold DNA, the biotin-unlabeled promoter of *xanfur*. 100×, 200×, and 400×, the cold DNA used to competitively inhibit the interaction between Clp and free DNA was 100-, 200-, or 400-folds of the free DNA concentration. The glutathione-S-transferase (GST) protein was used as the negative control. (**c**) The representative *in vitro* interaction between Clp and the 5-carboxyfluorescein-labeled promoter of *xanfur* by using microscale thermophoresis. The value of *K*_d_ represents their binding capacity. The GST protein was used as the negative control. The experiment was independently repeated three times. Values are the means ± SDs from three independent experiments.

To explain how Clp positively regulated the expression of *xanfur* in *Xoo*, we analyzed the interaction between the *xanfur* promoter and the Clp protein *in vivo* and *in vitro*. The *in vivo* results of B1H (bacterial one-hybrid) showed that all the *Escherichia coli* cells containing pTRG/pBXcmT-*xanfur*, pTRG-*clp*/pBXcmT-*xanfur,* or pTRG-R3133/pBXcmT-R2031 grew well on the M9-based medium plate without 3-amino-1,2,4-triazole (3-AT) and streptomycin. However, on the M9-based medium plate with 3-AT and 8 µg/mL streptomycin, the *E. coli* cells containing pTRG-*clp*/pBXcmT-*xanfur* and those containing pTRG-R3133/pBXcmT-R2031 grew well, but those containing pTRG/pBXcmT-*xanfur* failed to grow (Fig. 7a), suggesting that Clp can interact with the promoter of *xanfur*.

To verify the result of B1H, we purified the GST (glutathione-S-transferase) protein, and the GST-tagged Clp protein (Clp-GST), and analyzed their interactions with the free DNA (biotin-labeled promoter of *xanfur*) and the cold DNA (biotin unlabeled promoter of *xanfur*) by using EMSA (electrophoretic mobility shift assay). The result showed that while the band of complex representing the combination of the GST protein with the free DNA was not detectable, the band of complex representing the combination of the Clp-GST protein with the free DNA was detected and exhibited remarkable increase with an increased application of the Clp-GST protein from 0 µM to 0.15 µM. Meanwhile, the band of the free DNA exhibited remarkable decrease with the increased application of the Clp-GST protein. In addition, when excess cold DNA was present, the band of complex representing the combination of the Clp-GST protein with the free DNA exhibited remarkable decrease and the band of free DNA exhibited remarkable increase with an increased application of the cold DNA (100-, 200-, and 400-folds of free DNA) (Fig. 7b). Moreover, the interaction between Clp and the promoter of *xanfur* was confirmed by using MST (microscale thermophoresis), in which Clp-GST was found to bind to the promoter of *xanfur* with a high affinity (*K*_d_, 0.687 ± 0.0491 nM). In contrast, no affinity between GST and the promoter of *xanfur* was found (Fig. 7c). These results further demonstrated that Clp can bind the promoter region of *xanfur*.

Studies have reported that Clp can up-regulate the expression of enzyme genes related to the virulence of *X. campestris* pv. *campestris*, including *pmeA* encoding a pectin methylesterase, *manA* encoding a endo-1,4-β-mannosidase, and *pelA1* encoding a pectate lyase (39 – 41). In addition, it has been reported that Clp regulated the expression of virulence-related genes by binding to their promoter regions, such as *hshB* encoding a hydrolase in *X. oryzae* pv. *oryzicola* (42). Studies also have reported that the Clp binding site exhibits perfect twofold sequence symmetry, such as the 5´-ATCC-N8-GGAT-3´ and 5´-ATCG-N8-CGAT-3´ motifs of the heat-stable antifungal factor (HSAF) biosynthesis operon in *Lysobacter enzymogenes*, the 5´-CAC-N8-GTG-3´ motif of *PXO_03177* which is a virulence-related gene in *Xoo* (37, 43). Our results in this study showed that Clp also positively regulated the expression of *xanfur* by binding to its promoter region, but there is no 5´-ATCC-N8-GGAT-3´, 5´-ATCG-N8-CGAT-3´, or 5´-CAC-N8-GTG-3´ motif found in the promoter region. Then a 5´-TGGG-CAAGGTCG-CCCA-3´ motif was found in the promoter region of *xanfur*, exhibiting 93.75% similarity to the Clp binding site (5´-TGGG-CAAGGTGG-CCCA-3´) of *zur* gene in *X. campestris* pv. *campestris* (44). This indicated that the 5´-TGGG-N8-CCCA-3´ motif maybe the potential Clp binding site. Thus, it seems that Clp regulates a variety of virulence-related genes through different binding sites in pathogenic *Xanthomonas* species.

### Conclusion

Although *Xoo* has been found to be a bacterial pathogen causing bacterial leaf blight in rice for many years, the molecular mechanisms of the rice-*Xoo* interaction has not been fully understood. In this study, we found a novel Fur protein, XanFur, conserved in pathogenic *Xanthomonas* species, which is required for the full virulence of *Xoo* in rice. Moreover, exterior H_2_O_2_ significantly induces the expression of *xanfur* in *Xoo*, and *xanfur* is essential for the tolerance of *Xoo* to H_2_O_2_, suggesting that XanFur is likely involved in the response and resistance of *Xoo* to the oxidative stress caused by H_2_O_2_ in rice. *xanfur* is also essential for some important virulence determinants of *Xoo,* including EPS production, biofilm formation, and cell motility, further demonstrating that XanFur is likely involved in regulating the virulence of *Xoo*. In addition, it was found that Clp positively regulates the expression of *xanfur* by directly binding to its promoter region, especially in the presence of H_2_O_2_. These findings not only contribute to a better understanding of the interaction mechanisms between rice and *Xoo*, but also provide reference to developing high-effective bactericides by targeting to the Fur protein in *Xoo* to control bacterial leaf blight in rice.

## MATERIALS AND METHODS

### Strains, plasmids, and culture conditions

The bacterial strains and plasmids used in this study were listed in Table S1. The WT *Xoo* strain PXO99^A^ was preserved in our lab, which was named strain WT *Xoo* in this study. The *clp* deletion mutant strain of WT *Xoo* was constructed previously in our lab, which was named strain Δ*clp* in this study. The *xanfur* deletion mutant strains of WT *Xoo* and Δ*clp* were constructed in this study, which were named strains Δ*xanfur* and Δ*xanfur&clp*, respectively. The Δ*xanfur* complementary strains of Δ*xanfur* and of Δ*xanfur&clp* were constructed in this study, which were named strains CpΔ*xanfur* and CpΔ*xanfur*(Δ*clp*), respectively. All these strains were grown in NB liquid medium or MMX liquid medium or on NB solid medium at 28°C (45). *Escherichia coli* was grown in Luria-Bertani (LB) medium or on M9-based medium at 37°C (43). Antibiotics of kanamycin (50 µg/mL), spectinomycin (25 µg/mL), and gentamicin (25 µg/mL) were added to the growth medium as appropriate for selection.

### Bioinformatic analysis of XanFur

The conserved domain of XanFur in *Xoo* was analyzed by using the online software at the SMART website (http://smart.embl-heidelberg.de/). BLASTP was used to search for homologs of XanFur in *Xanthomonas* species and other pathogens from the website of National Center for Biotechnology Information. A total of 19 Fur proteins were used for pylogenetic analysis, including those in *Xoo* PXO99^A^ (WP011408024), *Xoo* BXO43 (AFF146830), *Xoo* KACC10331 (AAW74759), *X. campestris* pv. *azadirachtae* (OOX25098), *X. phaseoli* pv. *syngonii* LMG 9055 (OQP80446), *X. campestris* pv. *nigromaculans* (MCC4626846), *X. axonopodis* pv. *passiflorae* (MBV6814796), *X. arboricola* (WP_115047450), *X. oryzae* pv. *oryzicola* BLS256 (WP_002804358), *X. campestris* pv. *phaseoli* (AF146829), *X. campestris* pv. *campestris* (AF146020), *X. campestris* pv. *translucens* (AF146021), *X. campestris* pv. *vesicatoria* str. 85–10 (CAJ23192), *Chromobacterium violaceum* ATCC 12472 (WP_011135349), *Salmonella enterica* subsp. *enterica* serovar Typhimurium str. LT2 (NP_459678), *A. citrulli* AAC00-1 (ABM35092), *Pseudomonas aeruginosa* PAO1 (AAC05679), *P. aeruginosa* PA103 (AAA25820), and *Haemophilus influenzae* 86–028NP (AAX87247). The pylogenetic analysis was conducted in MEGA7 (46). Multiple-amino-acid sequence alignment was carried out by using ClustalW program (47).

### Generation of the gene *xanfur* deletion mutant and complementary strains

Generation of the in-frame deletion mutants of gene *xanfur* was carried out by using the strains WT and Δ*clp* as the parental strains via allelic homologous recombination, according to the previously described protocol (45). Here, two flanking regions were amplified by PCR using the corresponding primer pairs (Table S2), which then were digested with the related restriction enzymes, and were integrated into plasmid pK18mobsacB. The integrated plasmid was electroporated into the strains WT and Δ*clp*. Transformants were selected on NB solid medium plates containing 50 µg/mL kanamycin but without sucrose for the first cross-over event. The well-grown colonies were further cultured on nutrient broth with agar (NA) solid medium plate containing 10% (wt/vol) sucrose to obtain cells undergoing the secondary cross-over event. After two rounds of recombination, the resulting *xanfur* deletion mutant strains, Δ*xanfur* and Δ*xanfur&clp*, were confirmed by using PCR. For complementation, the gene *xanfur* with its predicted promoter region was amplified by using PCR with specific primers (Table S2), and then was cloned into plasmid pUFR047. The integrated plasmid was electroporated into the mutant strains Δ*xanfur* and Δ*xanfur&clp* to generate the complemented strains CpΔ*xanfur* and CpΔ*xanfur*(Δ*clp*), respectively.

### Bacterial growth assays in NB liquid medium and MMX liquid medium

The *Xoo* strains [WT, Δ*xanfur*, Δ*clp*, Δ*xanfur&clp*, CpΔ*xanfur,* and CpΔ*xanfur*(Δ*clp*)] were cultured in NB liquid medium to OD_600_ = 1.0, and then 500 µL bacterial suspensions were transferred into 250 mL flasks containing 50 mL sterilized NB liquid medium, which were incubated at 28℃ with shaking at 220 rpm. The value of OD_600_ of the bacterial suspensions was recorded every 2 h for 36 h. In addition, the cells of the *Xoo* strains were collected after the centrifugation at 6,000 g for 10 min when OD_600_ of the bacterial suspensions were 1.0. Then, the collected cells were suspended with MMX liquid medium to OD_600_ = 1.0, and 5 mL bacterial suspensions were transferred into 250 mL flasks containing 50 mL sterilized MMX liquid medium, which were incubated at 28℃ with shaking at 220 rpm. The value of OD_600_ of the bacterial suspensions was recorded every 8 h for 5 days. Each treatment was repeated at least three times.

### Virulence analysis in rice

The virulence of *Xoo* in rice was analyzed by using the leaf-clipping method (45). Briefly, the susceptible rice cultivar IR24 was planted in a growth chamber under a cycle of 16 h of light at 28°C and 8 h of dark at 25°C. The *Xoo* strains [WT, Δ*xanfur*, Δ*clp*, Δ*xanfur&clp*, CpΔ*xanfur,* and CpΔ*xanfur*(Δ*clp*)] were cultured in NB liquid medium to OD_600_ = 0.1, and then the cells of the *Xoo* strains were collected after the centrifugation at 6,000 g for 10 min. Then the collected cells were suspended with ddH_2_O to OD_600_ = 0.1, which were used for inoculation. Five-week-old rice leaves were inoculated with the bacterial suspensions by using scissors. Lesion lengths of the inoculated rice leaves were measured after 14 days. Meanwhile, 1 cm^2^ rice leaves were clipped from the inoculation sites and were ground in a 10 mL tube with 5 mL sterile distilled water. The ground suspensions were diluted 10^5^-folds, and 100 µL diluted suspensions were coated on NB solid medium plates, which were cultured at 28°C for 3 days. Then, the population of the *Xoo* strains (WT, Δ*xanfur,* and CpΔ*xanfur*) on NB solid medium plates were investigated. Fifty rice leaves were inoculated and four of them were clipped in each treatment. Each treatment was repeated at least three times.

### Determination of the tolerance to H_2_O_2_

The tolerance of *Xoo* to H_2_O_2_ was determined with the method described previously (37). Briefly, the *Xoo* strains (WT, Δ*xanfur,* and CpΔ*xanfur*) were cultured in NB liquid medium to to OD_600_ = 1.0, and then 80 µL bacterial suspensions were transferred into 8 mL sterilized NB liquid medium containing 0 mM, 0.1 mM, or 0.2 mM H_2_O_2_, which were incubated at 28°C with shaking at 220 rpm. The growth of the *Xoo* strains was observed every 12 h after incubation for 2 days. Meanwhile, the bacterial suspensions were diluted fivefolds (OD_600_ = 0.2) or 25-folds (OD_600_ = 0.04), and then 3 µL diluted bacterial suspensions were dropped onto the surface of NB solid medium plates containing 0 mM, 0.1 mM, or 0.2 mM H_2_O_2_, which were incubated at 28°C without shaking. The growth of *Xoo* strains were observed after incubation for 2 days. Each treatment was repeated at least three times.

### Measurement of EPS production

The EPS production of *Xoo* was measured with the method described previously (48). Briefly, the *Xoo* strains [WT, Δ*xanfur*, Δ*clp*, Δ*xanfur&clp*, CpΔ*xanfur,* and CpΔ*xanfur*(Δ*clp*)] were cultured in NB liquid medium to OD_600_ = 1.0, and then 500 µL bacterial suspensions were transferred into 250 mL flasks containing 50 mL sterilized NB liquid medium, which were incubated at 28°C with shaking at 220 rpm. After 5-day incubation, the bacterial suspensions were centrifuged at 12,000 g for 10 min. The collected supernatants of the *Xoo* strains were mixed with 100 mL ethanol, and incubated at room temperature for 24 h to precipitate the EPS. The precipitated EPS was dried at 70°C, and then weighed using a digital analytical balance. Each treatment was repeated at least three times.

### Biofilm formation assay

The biofilm formation of *Xoo* was measured with the method described previously (48). Briefly, the *Xoo* strains [WT, Δ*xanfur*, Δ*clp*, Δ*xanfur&clp*, CpΔ*xanfur,* and CpΔ*xanfur*(Δ*clp*)] were cultured in NB liquid medium to OD_600_ = 1.0, and then 40 µL bacterial suspensions were transferred into sterilized polystyrene tubes containing 4 mL NB liquid medium and incubated at 28°C for 24 h with shaking at 220 rpm. The polystyrene tubes were subsequently kept in a growth chamber at 28°C for 7 days without shaking, and then were gently washed three times with water after discarding the bacterial suspensions. The biofilm formed on the wall of the polystyrene tubes was stained with 0.1% crystal violet for 20 min. After that, the stained biofilm on the polystyrene tubes was dissolved in 4 mL acetic acid/ethanol (1:4, vol/vol), and the value of OD_590_ of the solutions were measured by using an Agilent 8453 UV-visible spectrophotometer (Agilent Technologies, USA). The value of OD_590_ was used as an indicator of the production of biofilm.

The 3D structure of bacterial biofilm was analyzed by using confocal laser scanning microscope, according to the previously described protocol (49). The plasmid pUFZ75 previously constructed for the expression of the green fluorescent protein (GFP) (50) was electroporated into the *Xoo* strains (WT and Δ*xanfur*). The GFP-labeled *Xoo* strains were cultured in NB liquid medium with 10 mM kanamycin to OD_600_ = 1.0, and then 200 µL bacterial suspensions were transferred into the flow chambers covered with glass slides. The flow chambers were kept in a humidified condition at 28°C for 48 h without shaking. Biofilm formation was visualized by using a confocal laser scanning microscope (Leica Microsystems Inc., USA) with an excitation wavelength of 488 nm and an emission wavelength of 500 to 545 nm. The images were analyzed by using the software of LAS_X_Small_2.0.0_14332. Each treatment was repeated at least three times.

### Measurement of cell motility

The cell motility was measured with the method described previously (48). The *Xoo* strains (WT, Δ*xanfur*, Δ*clp*, Δ*xanfur&clp*, CpΔ*xanfur* and CpΔ*xanfur*(Δ*clp*)) were cultured in NB liquid medium to OD_600_ = 1.0, and then 3µL bacterial suspensions were dropped onto the surface of NB solid medium plates containing 0.6% (wt/vol) agar (for swarming motility) or 0.3% (wt/vol) agar (for swimming motility). The swarming zones and swimming zones were calculated after the incubation of the *Xoo* strains on the plates at 28°C for 2 or 3 days without shaking. The calculated zone was used as an indicator of the cell motility. Each treatment was repeated at least three times.

### qRT-PCR analysis

The mRNA level expression of gene in bacteria was measured with the method described previously (51). The *Xoo* strains (WT and Δ*clp*) were cultured in NB liquid medium to OD_600_ = 1.0, and then 80 µL bacterial suspensions were transferred into 8 mL sterilized NB liquid medium with or without 0.1 mM H_2_O_2_ and incubated at 28°C for 24 h with shaking at 220 rpm. The cells of the *Xoo* strains were collected by centrifugating at 10,000 g for 10 min. Total RNA was extracted from the collected *Xoo* cells by using the TRIzol reagent (ThermoFisher, USA), and cDNA was synthesized from 100 ng total RNA by using the TransScript All-in-One First-Strand cDNA Synthesis SuperMix Kit (TransGen Biotech, China). qRT-PCR was performed in a QuantStudio 6 Flex Real-Time PCR System (Applied Biosystem, USA). The gene relative expression ratio was calculated by the method of 2^-ΔΔCt^. The 16S rRNA gene was used as the endogenous control. Each treatment was repeated at least three times.

### Western blot analysis

Western blot analysis of the protein expression in *Xoo* was performed with the method described previously (37). Briefly, the Flag tag (GAT TAC AAG GAT GAC GAC GAT AAG) was fused to the C terminus of gene *xanfur*, and then the integrated fragment was cloned into the plasmid pUFR047 which was previously constructed (52). The integrated plasmid pUFR047-*xanfur*-Flag was electroporated into the strains of *Xoo* (WT and Δ*clp*). Then, the cells of the Flag tagged *Xoo* strains were cultured in 50 mL sterilized NB medium with or without the presence of 0.1 mM H_2_O_2_ to OD_600_ = 1.0, and then the cells were collected by centrifugation at 6,000 g for 10 min. The total proteins were extracted from the collected cells by using 1 mL radioimmunoprecipitation assay lysis buffer containing 3 µL phenylmethanesulfonyl fluoride and 3 µL protease inhibitor cocktail. To obtain the soluble proteins, the supernatant was harvested after centrifugation at 10,000 g for 10 min. The soluble proteins were separated by SDS-PAGE and immobilized onto a polyvinylidene difluoride membrane by using the semi-dry blot machine (Bio-Rad, USA). The membrane was incubated in the blocking buffer containing the anti-Flag mouse primary antibody (1:5,000, Abmart, China), followed by an incubation in new blocking buffer containing the horseradish peroxidase (HRP)-conjugated goat anti-mouse secondary antibody (1:10,000, Abmart, China). After that, the membrane was stained using the Super ECL Plus kit and imaged using FluorChem M (Alpha Innotech, USA). The α-subunit of RNA polymerase was used as a control for sample loading. Each treatment was repeated at least three times.

### Protein expression and purification

Protein expression and purification were performed with the method described previously (43). Briefly, the coding region of *clp* was amplified from *Xoo* by PCR, and then was cloned into the plasmid pGEX-6P-1 which was previously constructed (53). The integrated plasmid pGEX-6p-1-Clp was transformed into *E. coli* strain BL21 (DE3) by heat shock for protein expression. The transformed strain was cultured in 400 mL LB liquid medium in the presence of 100 µg/mL ampicillin at 37°C with shaking at 220 rpm to OD_600_ = 0.4. Then, isopropyl β-D-1-thiogalactopyranoside at a final concentration of 0.4 mM was added to the LB medium, followed by a further incubation at 28°C with shaking at 220 rpm for 4 h. The *E. coli* cells were then lysed by sonication in 30 mL phosphate-buffered saline (PBS) lysis buffer containing 1 mM phenylmethanesulfonyl fluoride. The crude extraction was centrifuged at 13,000 g for 5 min. The collected supernatant was mixed with Glutathione Sepharose 4B (GE) at 4°C for 12 h. The Clp-GST protein was eluted using the elution buffer. The concentration of the purified Clp-GST protein was determined using a Bradford protein assay kit (Bio-Rad, USA).

### B1H assay

The *in vivo* interaction between the Clp protein and the promoter of *xanfur* was analyzed by using EMSA, according to the previously described protocol (43). The coding region of *clp* and the promoter region of *xanfur* was, respectively, cloned into plasmids pTRG and pBXcmT which were previously constructed (54). The two integrated plasmids pTRG-*clp* and pBXcmT-*xanfur* were transformed into the *E. coli* strain XL1-Blue MRF' kan by heat shock, and then the recombinant cells were cultured on M9-based medium plates containing 12.5 µg/mL tetracycline, 34 µg/mL chloramphenicol, 30 µg/mL kanamycin, 0 or 5 mM/L 3-amino-1,2,4-triazole and 0 µg/mL or 8 µg/mL streptomycin at 28°C. The well-grown cells indicated that direct physical interaction occurred between Clp and the promoter of *xanfur*. The cell of *E. coli* containing the plasimd pBXcmT-*xanfur* and the empty plasmid pTRG was used as the negative control. The cell of *E. coli* containing plasmids pBXcmT-R2031 and pTRG-R3133 was used as the positive control. Each treatment was repeated at least three times.

### EMSA assay

The *in vitro* interaction between the Clp protein and the promoter of *xanfur* was analyzed by using EMSA, according to the previously described protocol (43). The amplified biotin-labeled fragment of the *xanfur* promoter and the purified protein of Clp-GST were mixed and incubated according to the instruction of the LightShift EMSA Optimization & Control Kit (ThermoFisher, USA). The binding mixture was electrophoresed into the polyacrylamide gel, and then was transferred to a nylon membrane for nucleic acid blotting by using a semi-dry blot machine (Bio-Rad, USA). The biotinylated fragment of *xanfur* and its complex with Clp-GST protein on the membrane were detected by chemiluminescence using the VersaDoc imaging system (Bio-Rad, USA). The biotin-unlabeled fragment of the *xanfur* promoter and the purified GST protein were used as the negative controls. Each treatment was repeated at least three times.

### MST measurement

Binding of the Clp-GST fusion protein to the promoter of *xanfur* was determined by MST using Monolith NT.115 (NanoTemper Technologies, Germany), according to the previously described protocol (43). Briefly, FAM (5-carboxyfluorescein)-labeled fragment of the *xanfur* promoter was amplified by PCR. A constant concentration (10 µM) of the FAM-labeled *xanfur* promoter in the MST buffer (50 mM Tris pH 7.5, 150 mM NaCl, 10 mM MgCl_2_, 0.05% Tween 20) was titrated against increasing concentration of the purified Clp-GST protein dissolved in the MST buffer. MST premium-coated capillaries (Monolith NT.115 MO-K005, Germany) were used to upload the samples into the MST instrument at 25℃ using 40% MST power and 20% light-emitting diode (LED) power. The time of laser on was 30 s and laser off was 5 s. Data were analyzed by using Nanotemper Analysis software v.1.2.101 (NanoTemper Technologies, Germany). The purified GST protein was used as the negative control. The value of *K*_d_ represented the binding ability of Clp-GST or GST with the fragment of the *xanfur* promoter. Each treatment was repeated at least three times.

### Data analysis

All analyses were conducted by using SPSS 14.0 (SPSS Inc., USA). Significant differences were determined via the hypothesis test of percentages (*t*-test) (*P < 0.05; **P < 0.01).

## Data Availability

The original contributions presented in this study are included in the article/supplementary file, further inquiries can be directed to the corresponding authors. Additional supporting information can be found in the online version of this article on the publisher's website.

## SUPPLEMENTAL MATERIAL

Fig. S1 to S3 and Tables S1 and S2 spectrum.01187-23-s0001.pdf All the supplemental materials.

ASM does not own the copyrights to Supplemental Material that may be linked to, or accessed through, an article. The authors have granted ASM a non-exclusive, world-wide license to publish the Supplemental Material files. Please contact the corresponding author directly for reuse.
