# Supplementary material for: XanFur, a novel Fur protein induced by H2O2, positively regulated by the global transcriptional regulator Clp and required for the full virulence of Xanthomonas oryzae pv. oryzae in rice
Source: Microbiol Spectr. 2023 Oct 13;11(6):e01187-23. doi: 10.1128/spectrum.01187-23 (PMC10714925; doi:10.1128/spectrum.01187-23)
Supplement: Fig. S1 to S3 and Tables S1 and S2 — All the supplemental materials. [file spectrum.01187-23-s0001.pdf]

SUPPLEMENTARY MATERIALS

XanFur, a novel Fur family protein induced by H_2O_2, positively regulated by the global transcriptional regulator Clp and required for the full virulence of *Xanthomonas oryzae* pv. *oryzae* in rice

Yu-Qiang Zhang[1,2*] and Fengquan Liu[1*]

[1]Institute of Plant Protection, Jiangsu Academy of Agricultural Sciences, No. 50 Zhongling Street, Nanjing, Jiangsu, 210014, P. R. China

[2]State Key Laboratory of Microbial Technology, Shandong University, No. 72 Binhai Road, Qingdao, Shandong 266237, P. R. China

Key Words: bacterial leaf blight, *Xanthomonas oryzae* pv. *oryzae*, *xanfur*, virulence, Clp

***Correspondence author**:

Yu-Qiang Zhang: yqzh1989@sina.com; Fengquan Liu: fqliu20011@sina.com

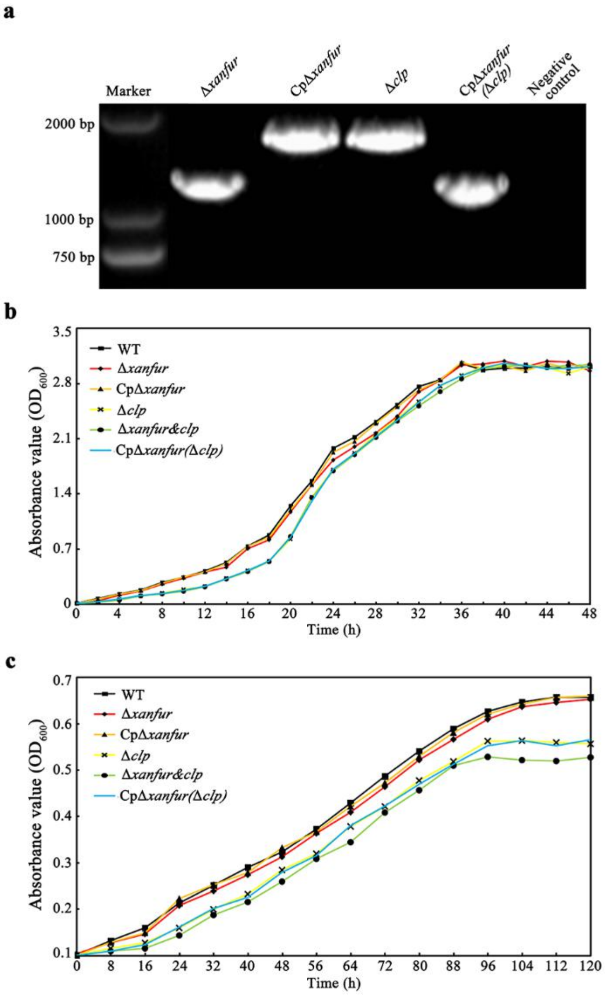

Fig. S1 Construction of the *xanfur* deletion mutants of *Xoo* strains WT and Δ*clp* and their complementary strains. (a) The representative *xanfur* deletion mutants of *Xoo* strains WT and Δ*clp* confirmed by using PCR. (b) The representative growth curves of *Xoo* strains WT, Δ*xanfur*, CpΔ*xanfur*, Δ*clp*, Δ*xanfur&clp* and CpΔ*xanfur(*Δ*clp)* which were cultured in NB liquid medium for 48 h. (c) The representative growth curves of *Xoo* strains WT, Δ*xanfur*, CpΔ*xanfur*, Δ*clp*, Δ*xanfur&clp* and CpΔ*xanfur(*Δ*clp)* which were cultured in MMX liquid medium for 120 h. WT, the wild-type *Xoo* strain PXO99^{A}. Δ*xanfur*, the *xanfur* deletion mutant strain of WT. CpΔ*xanfur*, the Δ*xanfur* complementary strain of WT. Δ*clp*, the *clp* deletion mutant strain of WT. Δ*xanfur&clp*, the *xanfur* deletion mutant strain of Δ*clp*. CpΔ*xanfur(*Δ*clp)*, the Δ*xanfur* complementary strain of Δ*clp*. The experiment was independently repeated three times.

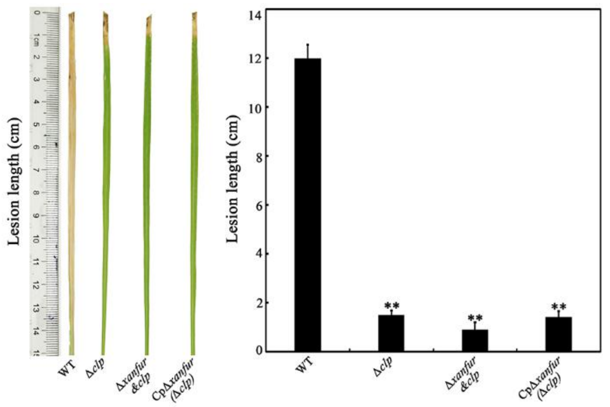

Fig. S2 The effect of *xanfur* deletion on the virulence of strain Δ*clp* in rice. The representative lesion length (left) and the calculated lesion length (right) in rice leaf 14 days post-inoculation of *Xoo* strains WT, Δ*clp*, Δ*xanfur&clp* and CpΔ*xanfur(*Δ*clp)*. WT, the wild-type *Xoo* strain PXO99^{A}. Δ*clp*, the *clp* deletion mutant strain of WT. Δ*xanfur&clp*, the *xanfur* deletion mutant strain of Δ*clp*. CpΔ*xanfur(*Δ*clp)*, the Δ*xanfur* complementary strain of Δ*clp*. The experiment was independently repeated three times. Values are the means ± SDs from three independent experiments. The asterisks above the error bars indicate significant differences compared with the wild-type strain (*t*-test, ***P* < 0.01).

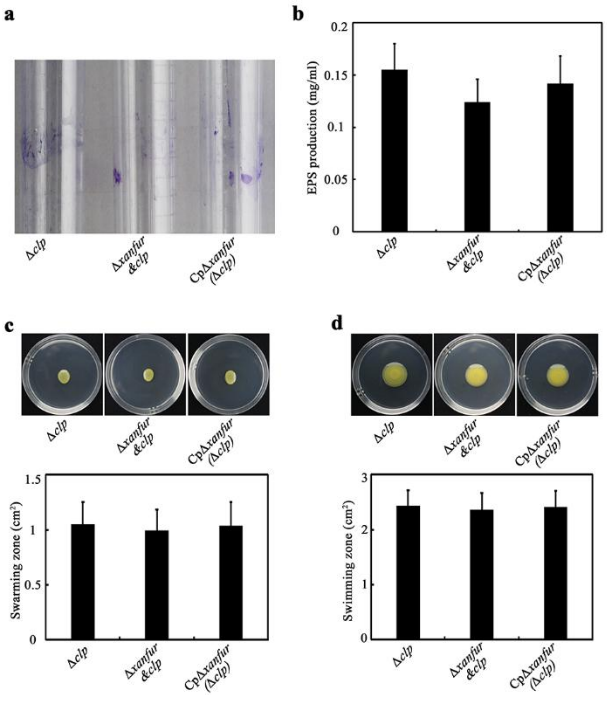

Fig. S3 The effect of *xanfur* deletion on the virulence determinants of strain Δ*clp*. (a) The representative biofilm formed by *Xoo* strains Δ*clp*, Δ*xanfur&clp* and CpΔ*xanfur(*Δ*clp)* on polystyrene tubes stained with crystal violet. (b) The calculated EPS production of *Xoo* strains Δ*clp*, Δ*xanfur&clp* and CpΔ*xanfur(*Δ*clp)*. (c) The representative swarming zone (up) and calculated area of swarming zone (down) produced by *Xoo* strains Δ*clp*, Δ*xanfur&clp* and CpΔ*xanfur(*Δ*clp)* which were cultured on NB solid medium plates containing 0.6% agar for 3 days. (d) The representative swimming zone (up) and calculated swimming zone (down) produced by *Xoo* strains Δ*clp*, Δ*xanfur&clp* and CpΔ*xanfu(*Δ*clp)* which were cultured on NB solid medium plate containing 0.3% agar for 2 days. Δ*clp*, the *clp* deletion mutant strain of wild-type *Xoo* strain PXO99^{A}. Δ*xanfur&clp*, the *xanfur* deletion mutant strain of Δ*clp*. CpΔ*xanfur(*Δ*clp)*, the Δ*xanfur* complementary strain of Δ*clp*. The experiment was independently repeated three times. Values are the means ± SDs from three independent experiments.

Table S1. Bacterial strains and plasmids used in this study.

**Strains and plasmids**	**Function**	**Source**
Xanthomonas oryzae **pv.** ***oryzae***		
PXO99^{A}	Philippine race 6	Lab collection
Δ*clp*	The gene *clp* deletion mutant in PXO99^{A}	Lab collection
Δ*xanfur*	The gene *xanfur* deletion mutant in PXO99^{A}	This study
CpΔ*xanfur*	The complementary strain of Δ*xanfur* in PXO99^{A}	This study
Δ*xanfur &clp*	The gene *clp* and *xanfur* double deletion mutant in PXO99^{A}	This study
CpΔ*xanfur*(Δ*clp*)	The complementary strain of Δ*xanfur* in Δ*clp*	This study

WT (pUFZ75)	WT harboring plasmid pUFZ75 (GFP-labeled strain); Km^R	This study
Δ*xanfur* (pUFZ75)	Δ*xanfur* harboring plasmid pUFZ75 (GFP-labeled strain); Km^R	This study
Escherichia coli		
DH5α		TransGen Biotech
XL1-Blue MRF´ Kan		Lab collection
BL21(DE3)		TransGen Biotech
**Plasmids**		
pUFR047	Broad-host-range expression vector	Lab collection
pGEX-6P-1	Plasmid used for protein expression	Lab collection
pTRG	Plasmid used for bacterial one-hybridization assay,	Lab collection
pBXcmT	Plasmid used for bacterial one-hybridization assay,	Lab collection
pK18mob*sacB*	Plasmid used for gene knockout	Lab collection
pUFR047-*xanfur*	pUFR047 carrying gene *xanfur* with its native promoter	This study
pGEX-6P-1-Clp	Plasmid used for the expression of Clp protein	This study
pTRG-*clp*	pTRG cloned with the intact *clp* gene	This study

pBXcmT-*xanfur*	pTRG cloned with the promoter of *xanfur*	This study
pK18- *xanfur*	pK18mob*sacB* with *xanfur* fragments	This study
pUFR047- *xanfur*	pUFR047 with gene *xanfur* and its native promoter	This study
pUFR047- *xanfur* -Flag	pUFR047 carrying gene *xanfur* with its native promoter region and Flag label; Gm^R, Amp^R	This study
pUFZ75	P_{trp}-TIR-gfp cassette in pUFR034, Km^R	Lab collection

Km^R, Gm^R and Amp^R, resistance to kanamycin, gentamicin and ampicillin, respectively.

Table S2. Primers used in this study.

Primer	Sequence (5' to 3')	Purpose/Description
**For mutant construction**		
xanfur -1F	CGGGATCCCGGCCGATGATTCTGGACGAGC	To amplify a 796 bp upstream homologue arm of *xanfur*
xanfur -1R	CGGAATTCCGTCGACGTGGTGATGCGGTGC	
xanfur -2F	CGGAATTCCGCGCTGGAAGTGCATGGGTT	To amplify a 591 bp downstream homologue arm of *xanfur*
xanfur -2R	GCTCTAGAGCCCTTGCCGGTGAAATACTCG	

For Complementation

CΔ *xanfur* -F	CGGGGTACCGCAGTGGACCAGTGG	To amplify a 1280 bp fragment containing encoding region of *xanfur* and its native promoter
CΔ *xanfur* -R	CGGAATTCCGTCGACGTGGTGATGCGGTGC	

For Bacterial one-hybrid assay

pBXcmT-*xanfur* -F	CGGAATTCGCAGTGGACCAGTGG	To amplify a 229 bp fragment containing the intact promoter of *xanfur*
pBXcmT-*xanfur* -R	GCTCTAGACATACGGGCTCCG	
pTRG-*clp*-F	CGGAATTCATCAGCTCCCTGCCGGCCTGC	To amplify a 693 bp fragment containing the intact *clp* gene
pTRG-*clp*-R	GCTCTAGAAACCGTCGTGTTTGCTGAGC	

For protein expression

pGEX-6P-1-*clp*-F	CCGCTCGAGGCGCGTGCCGTACAGCACGA	To amplify a 693 bp fragment containing the intact *clp* gene
pGEX-6P-1-*clp*-R	GGAATTCCATATGAGCTCAGCAAACACGACG	

For EMSA and MST

xanfur -F-biotin	GCAGTGGACCAGTGGAGCGTGG	To amplify a 229 bp fragment containing the intact promoter of *xanfur*
xanfur -R	ACGGGCTCCGGCGTCAGGCCGCG	
xanfur -F-FAM	GCAGTGGACCAGTGGAGCGTGG	To amplify a 229 bp fragment containing the intact promoter of *xanfur*
xanfur -R	ACGGGCTCCGGCGTCAGGCCGCG	
**For C-terminal Flag tag fusion in genome level**		
xanfur -Flag-F	CGGGGTACCGCAGTGGACCAGTGG	To amplify a 1304 bp upstream homologue arm of *xanfur* -Flag
xanfur -Flag-R	CGGAATTCCGTCGACGTGGTGATGCGGTGC	
**For qRT-PCR** (quantitative Real-time PCR)		
16S rRNA-F	TTCATGGAGTCGAGTTGCAG	The internal control for relative quantification
16S rRNA-R	GTCAAGTCATCATGGCCCTT	
qRT- *xanfur* -F	CACAAGCTCGAATCGGTCAA	To determine the transcriptional level of *xanfur*
qRT-*xanfur*-R	ATAACCCATGCACTTCCAGC	
